# Xeno-Free Condition Enhances Therapeutic Functions of Human Wharton’s Jelly-Derived Mesenchymal Stem Cells against Experimental Colitis by Upregulated Indoleamine 2,3-Dioxygenase Activity

**DOI:** 10.3390/jcm9092913

**Published:** 2020-09-10

**Authors:** Ji Yeon Kang, Mi-Kyung Oh, Hansol Joo, Hyun Sung Park, Dong-Hoon Chae, Jieun Kim, Hae-Ri Lee, Il-Hoan Oh, Kyung-Rok Yu

**Affiliations:** 1 Department of Medical Life Sciences, College of Medicine, The Catholic University of Korea, Seoul 08826, Korea; acme807@gmail.com (J.Y.K.); ipomk@catholic.ac.kr (M.-K.O.); so1hanssol@gmail.com (H.J.); hyunsungp@gmail.com (H.S.P.); chaedh96@naver.com (D.-H.C.); jieun_lyn@naver.com (J.K.); 2 Futuristic Animal Resource & Research Center, Korea Research Institute of Bioscience and Biotechnology, Chungcheongbuk-do 28116, Korea; 3 Catholic High-Performance Cell Therapy Center & Department of Medical Life Science, College of Medicine, The Catholic University, Seoul 08826, Korea; haeri1013@catholic.ac.kr; 4 Department of Agricultural Biotechnology and Research Institute of Agriculture and Life Sciences, Seoul National University, Seoul 08826, Korea

**Keywords:** stem cell therapy, mesenchymal stem cells, xeno-free medium, immunomodulatory property, indoleamine 2,3-dioxygenase (IDO), colitis model

## Abstract

The therapeutic applications of mesenchymal stem cells (MSCs) have been actively explored due to their broad anti-inflammatory and immunomodulatory properties. However, the use of xenogeneic components, including fetal bovine serum (FBS), in the expansion media might pose a risk of xenoimmunization and zoonotic transmission to post-transplanted patients. Here, we extensively compared the physiological functions of human Wharton’s jelly-derived MSCs (WJ-MSCs) in a xeno-free medium (XF-MSCs) and a medium containing 10% FBS (10%-MSCs). Both groups showed similar proliferation potential; however, the 10%-MSCs showed prolonged expression of CD146, with higher colony-forming unit-fibroblast (CFU-F) ability than the XF-MSCs. The XF-MSCs showed enhanced adipogenic differentiation potential and sufficient hematopoietic stem cell (HSC) niche activity, with elevated niche-related markers including CXCL12. Furthermore, we demonstrated that the XF-MSCs had a significantly higher suppressive effect on human peripheral blood-derived T cell proliferation, Th1 and Th17 differentiation, as well as naïve macrophage polarization toward an M1 phenotype. Among the anti-inflammatory molecules, the production of indoleamine 2,3-dioxygenase (IDO) and nitric oxide synthase 2 (NOS2) was profoundly increased, whereas cyclooxygenase-2 (COX-2) was decreased in the XF-MSCs. Finally, the XF-MSCs had an enhanced therapeutic effect against mouse experimental colitis. These findings indicate that xeno-free culture conditions improved the immunomodulatory properties of WJ-MSCs and ex vivo-expanded XF-MSCs might be an effective strategy for preventing the progression of colitis.

## 1. Introduction

Human mesenchymal stem cells (MSCs) have been isolated and expanded from many different adult and embryonic tissues [1]. MSCs are considered promising candidates for clinical applications in regenerative medicine because of their low immunogenicity and immunomodulatory capabilities for the treatment of a wide range of immune-related diseases, such as inflammatory bowel disease, graft-versus-host disease, and atopic dermatitis [2,3]. The immunomodulatory properties of MSCs involve soluble factors and cell contact-dependent processes in response to an inflammatory environment and immune cells [4]. MSC-mediated immunomodulation regulates both the innate and the adaptive immune system by inhibiting the proliferation of CD4 and CD8 T cells [5] and pro-inflammatory type 1 helper T cells (Th1) as well as interferon-gamma (IFN-γ) production by Th1 cells [6,7]. Furthermore, tumor necrosis factor (TNF)-α-mediated activation of MSCs promotes the polarization of naïve macrophages toward anti-inflammatory type (M2) macrophages and inhibits differentiation into a pro-inflammatory type phenotype (M1) and dendritic cells (DCs) [8,9]. We and others have shown that the soluble factors proposed to be involved during the immunomodulatory process include prostaglandin E2 (PGE2), indoleamine 2,3-dioxygenase (IDO), nitric oxide, transforming growth factor-beta (TGF-β), IL-6, and IL-10 [10,11].

Inflammatory bowel disease (IBD), including Crohn’s disease and ulcerative colitis, represents a group of pathogenic conditions characterized by chronic gastrointestinal tract inflammation. Although the etiology of IBD remains elusive, it is widely accepted that IBD results from an inappropriate response of the host’s immune system to intestinal microbes, possibly aggravated by genetic susceptibility [12]. Recent publications have shown the therapeutic potential of MSCs against IBD through immunomodulation, including the shift of pro-inflammatory immune response to a beneficial anti-inflammatory environment in the inflamed gastrointestinal tract [5,13]. Cellular interactions including the cell–cell contact, and paracrine effects between MSCs and immune cells cause suppression of B, T, and natural killer (NK) T cell proliferation as well as modulation of regulatory T (Treg) cells and macrophage activity, which leads to restoration of immune homeostasis [14,15].

Despite these possibilities for cell therapeutic applications of MSCs, in vitro culture of MSCs poses hurdles for the cellular function of MSCs. For example, continuous in vitro culture causes replicative senescence of MSCs, which is characterized by enlarged cell size, senescence-associated β-galactosidase expression, proliferation arrest, and compromised immunomodulatory properties [16,17]. Moreover, it has been shown that ex vivo expansion causes extensive heterogeneity of cellular function, which can originate from the distinct functional state of MSCs [18]. In addition, the use of fetal bovine serum (FBS), which has been frequently employed as a supplement for culture media during MSC culture, has other issues. The utilization of animal serum bears the risk of possible contamination with agents, such as viruses, bacteria, prions, mycoplasma, fungi, yeast, and endotoxins, that might be transmitted during MSC-based therapy [19,20].

This raises the necessity for developing an alternative to animal serum, mainly human blood-derived alternatives, such as autologous and allogeneic human serum albumin (HSA), human platelet lysates (hPL), and umbilical cord blood serum (UCBS) [21,22,23,24]. To provide a controlled and consistent environment for cell culture, chemically defined serum-free media has been developed for the expansion of MSCs, which contains a proprietary mix of proteins, such as human serum albumin, lipoproteins, recombinant human growth factors, and other components [25,26,27]. Cellular characteristic comparison studies showed similar surface marker expression, morphology, proliferation, and differentiation potential of MSCs cultured in media containing FBS vs. serum/xeno-free conditions [25,26]. However, only a few studies have characterized the immunomodulatory properties of MSCs in serum/xeno-free conditions. Adipose stem cells (ASCs) show stronger immunosuppression and lower immunogenicity in FBS culture conditions. However, by increasing the cell numbers and direct cell–cell contact, suppression can also be achieved in serum/xeno-free conditions [28]. More recently, Yoshida et al. reported the enhanced immunosuppressive and antifibrotic ability of MSCs cultured in serum-free conditions through elevated tumor necrosis factor-α-induced protein 6 (TSG-6) expression [29].

As one of the most promising cell therapy sources, currently, more than 1100 MSC-based clinical trials, either complete or ongoing, appear in the National Institutes of Health (NIH) database. However, the lack of standardization for the isolation, culture, and characterization of MSCs has complicated clinical interventions, often resulting in inconsistent outcomes. In a bid to achieve more predictable MSC-based therapy, serum/xeno-free media has been developed and used for MSC culture. Here, we extensively analyzed the functional properties of Wharton’s jelly-derived MSCs expanded in traditional, FBS-containing media, or serum/xeno-free-media conditions. Furthermore, we investigated the therapeutic efficacy of MSCs in different culture conditions in a murine model of colitis and examined the possible underlying mechanisms.

## 2. Materials and Methods

### 2.1. Preparation and Culture of WJ-MSCs

Wharton’s jelly-derived mesenchymal stem cells (WJ-MSCs) used in this study were isolated and cultured as previously described [30]. Briefly, Wharton’s jelly tissue was collected from the umbilical cords after a full-term, healthy delivery. The procedures for tissue harvest and obtaining informed consent were approved by the Asan Medical Center Institutional Review Board (protocol no. 2015–3030). Written informed consent was obtained from the parents. The MSCs were isolated from two Wharton’s jelly samples (WJ-MSC #1 and WJ-MSC #2). To analyze the effect of xenogeneic components of culture media on cell characteristics, WJ-MSCs were cultured in FBS and xeno-free (XF) conditions. For FBS conditions, Dulbecco’s Modified Eagle Medium (DMEM) (Gibco BRL, Grand Island, NY) was supplemented with 10% fetal bovine serum (Gibco), 1% Glutamax (Gibco), 1% antibiotics (Gibco), and basic fibroblast growth factor (bFGF) 10 ng/mL (Peprotech, Rocky Hill, NJ, USA). WJ-MSCs cultured in FBS conditions were detached by using 1% trypsin-EDTA (Gibco). For XF conditions, WJ-MSCs were seeded on vitronectin-coated cell culture flasks (Gibco) and cultured in STEMPRO MSC SFM XENOFREE (Gibco) supplemented with 1% Stempro MSC SFM Xeno-Free supplement, 1% Glutamax, and 1% antibiotics. WJ-MSCs cultured in XF conditions were detached by using TrypLE Select (Gibco). For long-term culture, the cells were seeded at 1 × 10^6^ cells in 100 mm culture dish, and passaging of MSCs was performed upon reaching 80–90% confluency.

### 2.2. Flow Cytometric Analysis of Immunophenotype

WJ-MSCs cultured in FBS and XF conditions were analyzed for surface markers by flow cytometry (BD FACSCantoⅡ™ Software, BD Biosciences, San Jose, CA, USA) using monoclonal antibodies against CD29-APC, CD44-PE, CD73-PE, CD90-FITC, CD105-APC, CD146- PECy7, CD34-BV421, and CD45-BV421 (BD biosciences).

### 2.3. In Vitro Differentiation Assay

For differentiation into adipocytes and osteoblasts, WJ-MSCs cultured in FBS and XF conditions were plated in 6-well plates at a concentration of 1 × 10^5^ cells. After the cells reached 70–80% confluency, they were treated with StemMACS™ Adipodiff Media and OsteoDiff Media (Miltenyi Biotec, Bergisch Gladbach, Germany), following the manufacturer’s instructions. The medium was changed every 3 days. After 3 weeks of induction, Oil Red O (Abcam, Cambridge, UK) staining or Alizarin Red S (Sigma-Aldrich, St. Louis, MO) staining was performed to evaluate lipid-laden fat cells or calcium deposition, respectively. For chondrogenic differentiation, 2.5 × 10^5^ cells were placed in a 15-mL polypropylene tube and maintained with 1 mL of StemMACS™ Chondrodiff Media (Miltenyi Biotec), following the manufacturer’s instructions. The cells were allowed to undergo differentiation for 3 weeks, with media change every 3 days. After differentiation, the round pellets were embedded in paraffin and cut into 3-µm sections that were stained by Alcian Blue (Merk Millipore, Darmstadt, Germany) to detect glycosaminoglycans.

### 2.4. Cumulative Population Doubling Level

The proliferation potential of WJ-MSCs cultured in FBS and XF conditions was determined by calculating the cumulative population doubling level (CPDL) in continual subculture and growth from a number of cells. The CPDL was calculated using the following equation: CPDL = ln (Nf/Ni) ln2, Ni is the initial cell number, Nf is the last cell number, and ln is the natural log. Then, 1 × 10^5^ cells were cultured (*n* = 3) on a 6-well plate, the number of cells was measured after 3 days, and 1 × 10^5^ cells were cultured again and repeatedly passaged. Calculated CPDL rates were added serially and represented as a broken line graph.

### 2.5. Isolation and Culture of Human Umbilical Cord Blood (hUCB)-Derived Mononuclear Cells (MNCs)

Umbilical cord blood (UCB) units were obtained from the Catholic Hematopoietic Stem Cell Bank (CHSCB) in Korea from April 2019 to June 2020 under the institutional review board’s approval (IRB No.2019-0467-0003). The UCB samples were mixed with HetaSep solution (Stem Cell Technologies, Vancouver, BC, Canada) at a ratio of 5:1. After incubation at room temperature for 1 h, the supernatant was carefully collected, and the mononuclear cells were obtained by Ficoll gradient centrifugation (Ficoll-Paque PLUS, GE Healthcare, Chicago, IL, USA) and resuspended in Roswell Park Memorial Institute (RPMI) 1640 medium (Gibco) supplemented with 10% FBS.

### 2.6. CFSE Proliferation Assay

WJ-MSCs were treated with 10 μg/mL of mitomycin C (MMC, Sigma) for 1 h to arrest cell proliferation. After 2 washes with PBS, WJ-MSCs were plated in a 96-well plate at 1 × 10^4^/well for 24 h. For the T cell proliferation assay, hMNCs were stained with CFSE using a CellTrace CFSE Cell Proliferation Kit (2 μM, Thermo Fisher Scientific, Waltham, MA, USA) according to the manufacturer’s instructions. hMNCs (1 × 10^5^) were added to wells containing MSCs, in the presence of anti-CD3/CD28 microbeads (Gibco) and recombinant human IL-2 (30 U/mL, PeproTech). Then, 6 days after co-culture, the cells were stained with fluorescence-labeled human monoclonal antibodies against CD3-BV510, CD4-APC, and CD8-BV421 (BD Biosciences) and T cell proliferation was measured for CFSE dilution by flow cytometry.

### 2.7. Hematopoietic Stem Cell (HSC) Expansion Analysis

The hUCB-derived MNC population was labeled with anti-CD34-conjugated microbeads (Miltenyi Biotec) according to the instructions of the manufacturer. CD34^+^ HSCs were enriched by magnetic cell separation using MACS columns (Miltenyi Biotec) and used immediately for co-culture experiments. CD34 + HSCs were co-cultured with 10%-MSCs or XF-MSCs in 12-well plates (ratio of cell number: MSCs:HSCs = 1 × 10^5^:1 × 10^4^). On day 6, HSCs were labeled with monoclonal antibodies against CD45-APC-H7, CD34-BV421, and CD90-FITC and analyzed by flow cytometry using FACSCantoⅡ™.

### 2.8. Generation and Stimulation of Macrophages

To induce differentiation to macrophages, THP-1 cells were pre-treated for 24 h with 100 nM phorbol 12-myristate 13-acetate (PMA, Sigma-Aldrich) and further incubated in fresh RPMI 1640 medium (Gibco) for 24 hours. On day 2, differentiated macrophage cells were stimulated with M1 cytokines (20 ng/mL IFN-γ, 1 μg/mL LPS; Peprotech) or M2 cytokines (20 ng/mL IL-4, 20 ng/mL IL-13; Peprotech), with or without WJ-MSCs, in a 12-well transwell plate (0.4 μM pore size, Corning, Lowell, MA, USA). WJ-MSCs were cultured at a density of 1X10^5^ in the upper layer, while THP-1 cells were placed at a density of 5 × 10^5^ in the lower layer in RPMI 1640 medium supplemented with 10% FBS. After co-culture for 48 h, the cells were stained with fluorescence-labeled human monoclonal antibodies against CD14-APC-H7, CD80-PE-Cy7, and CD163-BV421 (BD Biosciences) and analyzed by flow cytometry.

### 2.9. Th Cell Analysis

Human peripheral blood samples donated by healthy donors were provided from the Korean Red Cross Blood Services (Seoul, Korea) after obtaining informed consent. All experiments using human blood were conducted under approval of the Institutional Review Board (IRB) of the Catholic University (IRB No.2019-2891-0003). Peripheral blood was mixed with HetaSep solution for 1 h, the supernatant was placed on the Ficoll-Paque PLUS, and peripheral blood mononuclear cells (PBMCs) were separated by density-gradient centrifugation. To isolate CD4+ T cells, PBMCs were incubated with anti-CD4-conjugated microbeads (Miltenyi Biotec) for 30 min at 4 °C. The cells were separated on a magnetic field and then CD4+ cells were enriched by positive selection. For Th cell development, lineage-driving cytokine for Th1 (50 ng/mL IL-2, 25 ng/mL IFN-γ, 25 ng/mL IL-12; Peprotech), Th17 (50 ng/mL IL-6, 25 ng/mL TGF-β, 20 ng/mL IL-2; Peprotech) were added to CD4+ T cells in the presence of anti-CD3/CD28 microbeads. WJ-MSCs were treated with MMC (10 μg/mL) for 1 h and plated at 2.5 × 10^5^/well in a 24-well plate. After 24 h, cytokine-treated CD4+ T cells (5 × 10^5^/well) were co-cultured with WJ-MSCs in a 24-well plate. The cells were co-cultured for 5 days and stimulated with cell stimulation cocktail (Invitrogen) for 5 h then fixed/permeabilized (Invitrogen) and stained with CD4, IFN-γ (Th1), and IL-17A (Th17). The cells were analyzed by flow cytometry.

### 2.10. Cell Viability Assay

To assess the effect of inhibitors on PBMCs survival, PBMCs were stained with 2 μM CFSE and then seeded at a density of 1 × 10^5^ in a 96-well plate with or without treatment with COX-2 inhibitor celecoxib (0.05 μM or 0.1 μM, Sigma), IDO inhibitor 1-methyl-L-tryptophan (0.05 mM or 0.1 mM, Sigma), or NO inhibitor 1400W (0.05 mM or 0.1 mM Sigma) for 48 and 72 h. The cells were stained with 7-Aminoactinomycin D (7AAD) and analyzed by flow cytometry.

### 2.11. Western Blot Analysis

After treatment with recombinant human IFN-γ and TNF-α (Peprotech) for 24 h, the cells were lysed in RIPA buffer (Thermo Fisher Scientific) with protease inhibitor. Protein concentrations were quantified using BCA assay kit (Thermo Fisher Scientific). Equal amounts of protein were separated by 10% SDS-polyacrylamide gel and analyzed with the following human antibodies: anti-IDO (Merk millipore), anti-TGF-β1 (Abcam), anti-NOS2 (Santa cruz Biotechnology, Dallas, TX, USA), anti-TSG-6 (R&D systems, Minneapolis, MN, USA), and anti-GAPDH (GeneTex, Irvine, CA, USA). The proteins were detected using an enhanced chemiluminescence detection kit (Thermo Fisher Scientific) and luminescent image analysis LAS-3000 system (Fujifilm, Tokyo, Japan).

### 2.12. Quantitative PCR

Total RNA was isolated using TRIzol reagent (Invitrogen, Waltham, MA, USA) according to the manufacturer’s protocol and reverse-transcribed into cDNA using Superscript™ III reverse transcriptase (Invitrogen). Quantitative PCR was performed in a MX300P thermal cycler (Stratagene, San diego, CA, USA) using the SYBR Green Master Mix (Applied Biosystems, Foster City, CA, USA). The mRNA levels of each gene were normalized using GAPDH as housekeeping controls. At least three independent analyses were performed for each gene. Primer sequences are listed in Table 1.

### 2.13. CFU-F Assay

WJ-MSCs cultured in FBS and XF conditions were seeded in 100-mm culture dish at a density of 2 × 10^3^ or 3 × 10^3^ in growth medium. After 14 days of culture, the dishes were fixed with 100% methanol for 10 min and stained with 1% crystal violet solution (Sigma-Aldrich) for 30 min. The plates were then rinsed under distilled water and dried. The formation of colonies was observed.

### 2.14. Colitis Induction

All animal experiments were carried out in accordance with the approved guidelines of the Catholic University of Korea Institutional Animal Care and Use Committee (IACUC no. 2019-0301-03). Acute colitis in mice was induced by administration drinking water containing 3% (w/v) dextran sulfate sodium (DSS) for 7 days. Mice (*n* = 10 per group) were randomly assigned to the following groups: untreated control group (negative control), DSS treatment only (positive control), DSS+10%-MSCs administration, and DSS+XF-MSCs administration. Then, 10%-MSCs and XF-MSCs resuspended in PBS (2 × 10^6^ cells in 200µl) were injected intraperitoneally into mice 1 day after the administration of DSS. The mice were checked each day for morbidity and body weight was monitored for 11 days. On day 7, colitis severity was measured by utilizing the disease activity index (DAI), including body weight loss (0–4), stool consistency (0–4), bleeding (0–4), coat roughness (0–4), mouse activity (0–2), and bedding contamination by stool and blood (0–2). On day 11, the mice were sacrificed and their colon length and weight were measured. Histopathological evaluation was performed with the tissue.

### 2.15. Histopathologic Evaluation

Colon tissue samples were fixed in 4% formaldehyde (Wako, Richmond, VA, USA) and embedded in paraffin. Then, 5 µm thick sections were prepared from each block and stained with hematoxylin and eosin (H&E). Tissue sections were evaluated and observed under phase contrast microscope. Histological evaluation was performed in a blinded manner by scattered infiltration of inflammatory cells in the lamina propria and submucosa (0~4) and intestinal damage based on destruction of entire epithelium and severe submucosal edema (0~4).

### 2.16. Statistical Analysis

The experiments are shown as a summary of the data. At least three experiments were performed and presented as the mean ± S.D. Statistical analysis was performed using GraphPad Prism software (version 8.0.1; GraphPad Software, San Diego, CA, USA). Where data were normally distributed, the significance of the data was determined by using Student’s t-tests or one-way analysis of variance (ANOVA), followed by Bonferroni’s test analysis. For analysis of survival data, the Kaplan–Meier test was used. A p-value of less than 0.05 was considered to be statistically significant and is indicated in the text.

## 3. Results

### 3.1. Characterization of WJ-MSCs Cultured in FBS and XF Conditions

**Figure 1:**
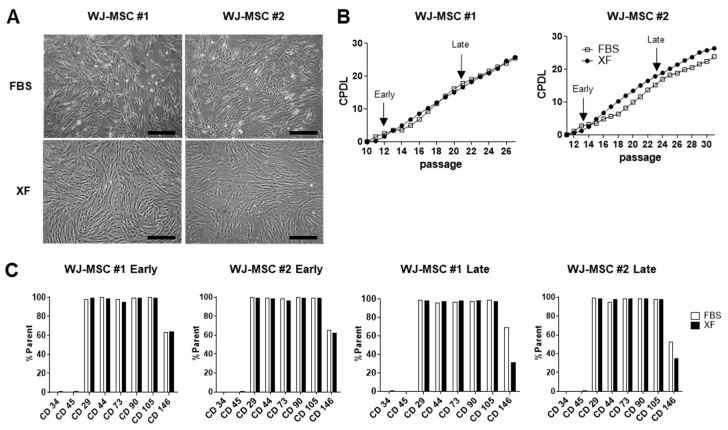
Characterization of Wharton’s jelly-derived MSCs (WJ-MSCs) cultured in 10% fetal bovine serum (FBS) and xeno-free medium (XF) conditions. (**A**) Morphologic images of human WJ-MSCs derived from two donors (WJ-MSC #1, WJ-MSC #2) cultured in FBS or XF conditions. Scale bar, 100 µm. (**B**) The cumulative population doubling levels (CPDL) were calculated to evaluate the proliferative ability of the WJ-MSCs cultured in FBS and XF conditions. Early passages and late passages were designated as 12–15 passages, 21–25 passages. (**C**) Immunophenotypes of WJ-MSCs cultured in FBS and XF conditions were measured by flow cytometry for the expression of MSC-specific antigens CD29, CD44, CD73, CD90, CD105, CD146 and HSC-specific antigens CD34, CD45.

The morphology of WJ-MSCs cultured in XF medium or 10% FBS medium was analyzed using phase-contrast microscopy. XF-MSCs exhibited small, spindle-like-shaped morphology, whereas 10%-MSCs had a flattened, fibroblast-like morphology (Figure 1A). A comparison of cell growth in 10% FBS and XF conditions showed no significant difference (Figure 1B). When the surface phenotype was compared, the characteristic WJ-MSC immune phenotypes of CD29, CD44, CD73, CD90, and CD105 were similarly expressed in XF and 10% FBS conditions, but the expression of CD146 was reduced in long-term expanded cells under XF conditions (Figure 1C and Supplementary Figure S1). To confirm the colony-forming unit-fibroblast (CFU-F) ability of 10%-FBS and XF-MSCs, the cells were plated at low density in a 100-mm dish. The XF-MSCs did not support colony formation, whereas the 10%-MSCs efficiently generated CFU-Fs (Supplementary Figure S2). These results indicate that the long-term culture of XF-MSCs did not affect proliferation or immunological phenotype but significantly reduced CFU-F ability.

### 3.2. WJ-MSCs Expanded in XF Conditions Show Increased Adipogenesis but Reduced Osteogenic Differentiation Potential

**Figure 2:**
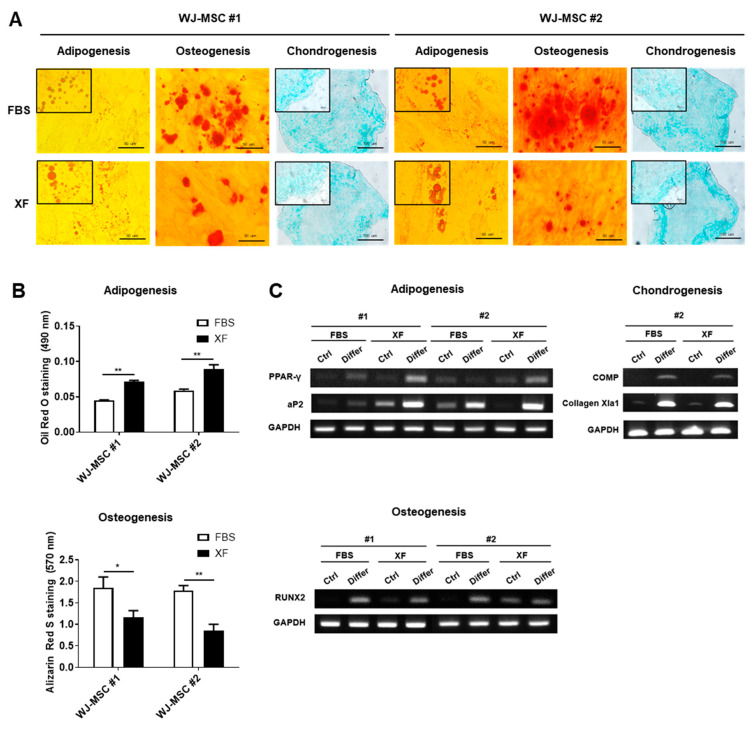
Differentiation potential of WJ-MSCs cultured in FBS and XF conditions. WJ-MSCs cultured in FBS and XF conditions were cultured with adipogenic, osteogenic, chondrogenic differentiation medium for 21 days. (**A**) Light microscopic images show representative adipocyte, osteocyte, and chondrocyte. The lipid droplets were visualized using Oil Red O staining on adipogenic cells and calcium phosphate deposits were stained with Alizarin Red S. Glycosaminoglycans of the chondrogenic differentiated cells were stained with Alcian Blue. Representative images from at least three independent experiments are shown. (**B**) Quantification of the released dye of Oil Red O staining and Alizarin Red S staining were measured by a spectrophotometer. Data are represented as the means ± S.D. of triplicate experiments (* *p* < 0.05, ** *p* < 0.01). (**C**) Expressions of adipogenic (PPAR-γ, aP2), osteogenic (RUNX2), and chondrogenic (COMP, Collagen Xla1) genes were determined by RT-PCR with GAPDH as the reference gene.

To test the differentiation potential of human WJ-MSCs expanded in FBS or XF conditions, the in vitro differentiation of adipogenic, osteogenic, and chondrogenic lineages was performed. After 21 days of differentiation induction, the adipogenic, osteogenic, and chondrogenic differentiation capacities of XF-MSCs and 10%-MSCs were examined by immunostaining with Oil Red O (lipid droplets), Alizarin Red (calcium phosphate deposits), and Alcian Blue (sulfated glycosaminoglycan) (Figure 2A). Under adipogenic induction conditions, accumulated lipid droplets were more efficiently formed, whereas the osteogenic induction confirmed by calcium phosphate deposition was downregulated in the XF-MSCs (Figure 2A,B). To quantify the extent of differentiation potential, we measured the expression levels of lineage-specific genes. XF-MSCs showed higher expression of PPAR-γ and aP2 compared to the 10%-MSCs. In contrast, the expression of RUNX2, a marker of osteogenic differentiation, was reduced in the XF-MSCs (Figure 2C). The chondrogenic differentiation potential between the XF-MSCs and 10%-MSCs was similar. These data suggest that XF culture conditions significantly increased the adipogenic but reduced the osteogenic differentiation capacity of WJ-MSCs.

### 3.3. XF Conditions Support the Hematopoietic Activity of WJ-MSCs

**Figure 3:**
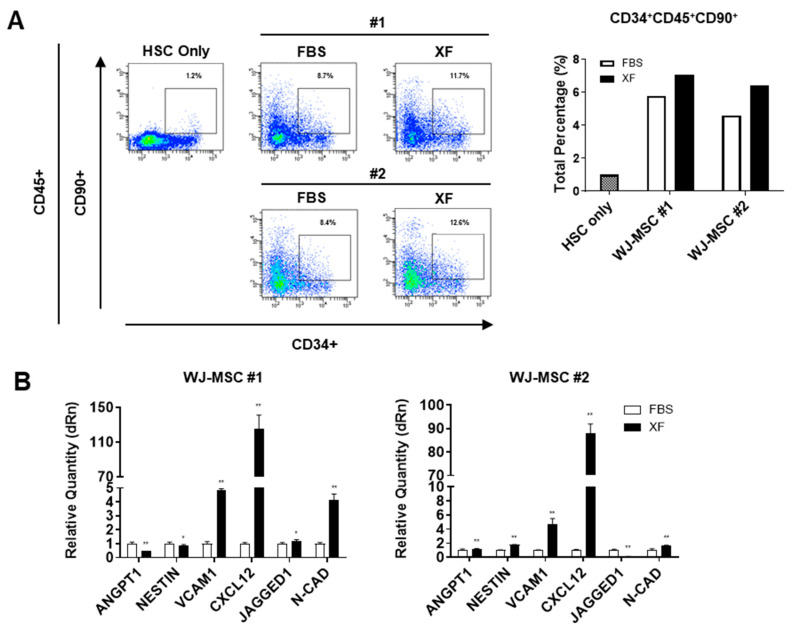
XF-MSCs support the human CD34+CD90+ HSC activity. (**A**) UCB-derived CD34+ HSC were co-cultured with 10%-MSCs or XF-MSCs for 6 days, and the percentage of CD45+, CD34+, CD90+ HSC subpopulations were measured using flow cytometry. The flow cytometry plots are representative of independent experiments performed at least three times. (**B**) mRNA expressions of HSC niche related genes in 10%-MSCs or XF-MSCs were determined by qRT-PCR and normalized to GAPDH (* *p* < 0.05, ** *p* < 0.01).

Next, we examined the effect of culture conditions on the ability of WJ-MSCs to support HSCs. We compared the HSC-supporting activity of WJ-MSCs by co-culturing UCB-derived human CD34+ hematopoietic progenitor cells with 10%-MSCs or XF-MSCs. The WJ-MSCs expanded under XF conditions supported the primitive compartment of hematopoietic progenitors, as manifested by an expansion of CD34+90+ cells, which are known as long-term repopulating hematopoietic cells [31], comparable to the FBS conditions (Figure 3A). As shown in Figure 3B, WJ-MSCs expanded under XF conditions exhibited higher expression levels of HSC niche-related genes, such as N-CAD and CXCL12, than in the FBS conditions. These results showed that WJ-MSCs could exert HSC-supporting activities as well as an activated niche state to support the maintenance of HSCs under XF conditions.

### 3.4. XF Condition Enhances the Immunosuppressive Properties of WJ-MSCs via IDO Production

**Figure 4:**
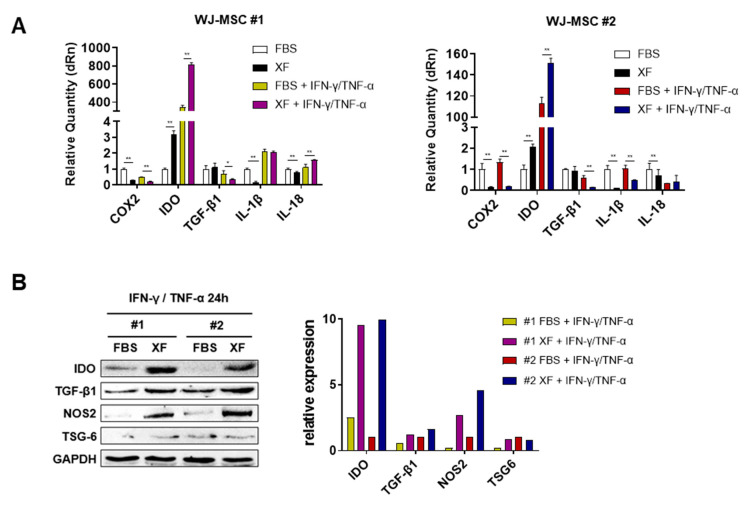
XF conditions enhance the expression of immunoregulatory factors in WJ-MSCs. WJ-MSCs were cultured in FBS and XF conditions were treated with or without IFN-γ (10 ng/mL) and TNF-α (10 ng/mL) for 24 h. (**A**) Gene expression of the immunoregulatory and inflammatory factors was determined by qRT-PCR (* *p* < 0.05, ** *p* < 0.01). (**B**) After IFN-γ and TNF-α treatment for 24 h, IDO, TGF-β1, NOS2, and TSG-6 expression were measured by Western blot analysis. The results are representative of independent experiments performed at least three times.

**Figure 5:**
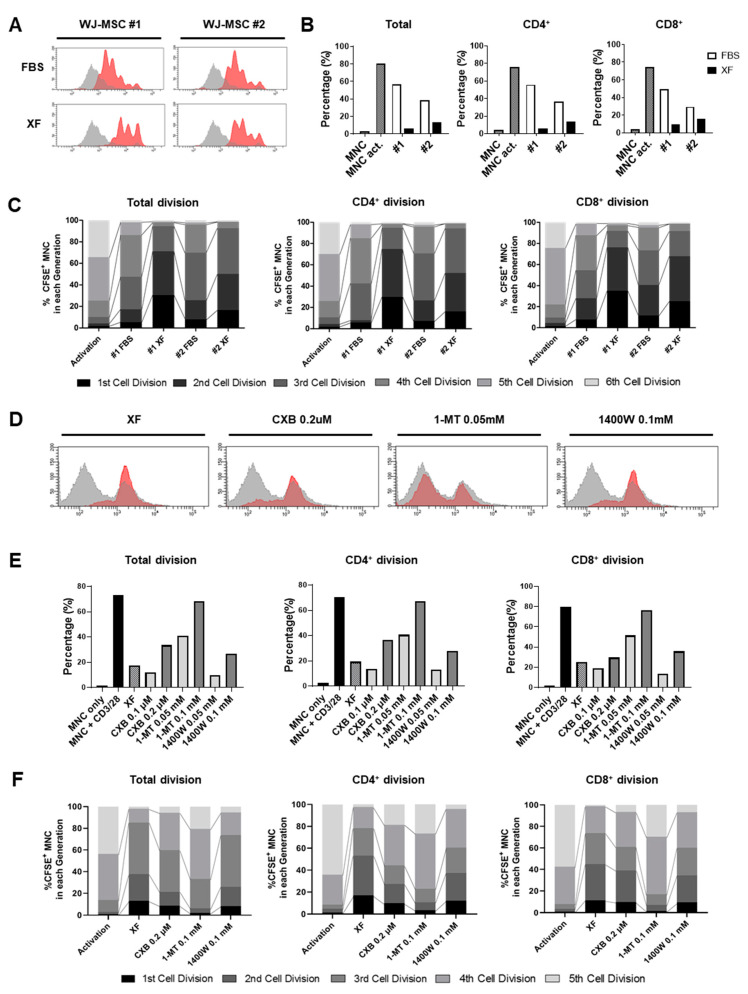
Long-term culture in XF conditions increases the immunomodulatory properties of WJ-MSCs. (**A**–**C**) Umbilical cord blood-derived mononucelar cells (UCB-MNCs) were stained with carboxyfluorescein succinimidyl ester (CFSE) and stimulated by anti-CD3/28 beads and IL-2 for T cell activation. Activated T cells were co-cultured with 10%-FBS or XF-MSCs. After 6 days, T cell proliferation was assessed in T subsets using flow cytometry. (**A**) Representative histogram of total T cell proliferation. (**B**) Proliferation rates of the total T, CD4+ T, and CD8+ T cells were quantified. (**C**) Cell divisions in the six distinct compartments were tracked using CFSE staining. (**D**–**F**) Peripheral blood mononuclear cells (PBMCs) were stained with CFSE and stimulated by anti-CD3/28 beads and IL-2 for T cell activation. After treating XF-MSCs with celecoxib, 1-MT, or 1400W at the indicated dose, the cells were incubated with activated T cells (PBMCs donor#1). A representative histogram of total T cell proliferation is shown (**D**), and the proliferation rate of the total T, CD4+ T, and CD8+ T cells was quantified (**E**). (**F**) Cell divisions in the five distinct compartments were tracked using CFSE staining. The results are representative of independent experiments performed at least three times.

The expression of COX2, IDO, TGF-β1, IL-1β, and IL-18 was measured to evaluate the immunomodulatory ability of WJ-MSCs expanded in FBS or XF conditions. It has been reported that the immune response of MSCs is elicited by pro-inflammatory cytokines [5]. Therefore, the WJ-MSCs were primed with TNF-α and IFN-γ. Each independent line of WJ-MSCs expanded under XF conditions showed significantly higher expression levels of IDO than the 10%-MSCs in the presence or absence of TNF-α and IFN-γ. However, the COX-2 mRNA levels were slightly reduced in the XF-MSCs in the presence of TNF-α and IFN-γ, indicating that COX-2/PGE-2 signaling might not be the main immunomodulatory pathway of the XF-MSCs. The expression of pro-inflammatory cytokines, such as IL-1β and IL-18, was slightly reduced in the XF-MSCs compared to the 10%-MSCs (Figure 4A). We then confirmed the protein expression levels of immunomodulatory molecules of WJ-MSCs expanded under XF and FBS conditions after TNF-α and IFN-γ activation. Consistent with the mRNA expression results, the IDO protein level was significantly increased in the XF-MSCs. In addition, the protein expression of TGF-β1 and NOS2 was increased in the XF-MSCs compared to the 10%-MSCs (Figure 4B). Next, we assessed the immunosuppressive properties of WJ-MSCs expanded in FBS or XF conditions through a co-culture assay of carboxyfluorescein succinimidyl ester (CFSE)-loaded human umbilical cord blood-derived mononuclear cells (hUCB-MNCs) or human peripheral blood mononuclear cells (PBMCs) and WJ-MSCs. When in vitro CD3/28 and IL-2-stimulated lymphocytes were cultured with XF-MSCs, the total T, CD4+ T, and CD8+ T cell proliferation rate was more suppressed compared to that of the 10%-MSCs (Figure 5A,B). A total of six cell divisions were detected by CFSE fluorescence. The percentage of cells in the initial division cycles 1 and 2 was significantly higher in the presence of XF-MSCs, whereas the percentage of cells in division cycles 4, 5, and 6 was significantly reduced (Figure 5C and Supplementary Figure S3A), suggesting that the proliferation of T cells was more effectively suppressed in XF-MSCs. To determine the key immunomodulatory factor of XF-MSCs, an inhibition assay was performed using the COX-2 inhibitor celecoxib, the IDO inhibitor 1-methyl-L-tryptophan (1-MT), and the NO inhibitor 1400W. The suppressed total T, CD4+ T, and CD8+ T cell proliferation in the XF-MSCs was reversed to positive control levels in the presence of high-dose 1-MT (Figure 5D), and a similar pattern was confirmed in another XF-MSC line (Supplementary Figure S4A). Celecoxib and 1400W also partially reversed the inhibited T cell proliferation at high concentrations, but the rate in these activated cells did not reach that of the positive control (Figure 5E). Cytokine inhibitors did not significantly alter the proliferation and cell viability of the PBMCs, suggesting that the inhibitors worked through MSCs but did not directly affect PBMCs (Supplementary Figure S4B). In the inhibitor study, a total of five cell divisions were detected by CFSE fluorescence, and the increased percentage of cells in the initial division cycles 1, 2, and 3 in XF-MSCs was reduced in the presence of high doses of 1-MT (Figure 5F and Supplementary Figure S3B), suggesting that the immunosuppressive function of IDO inhibitor-treated XF-MSCs was significantly inhibited. Taken together, these results indicate that enhanced IDO expression and function are the main T cell suppressive mechanisms of XF-MSCs.

### 3.5. XF-MSCs Regulate Helper T Cell and Macrophage Polarization

**Figure 6:**
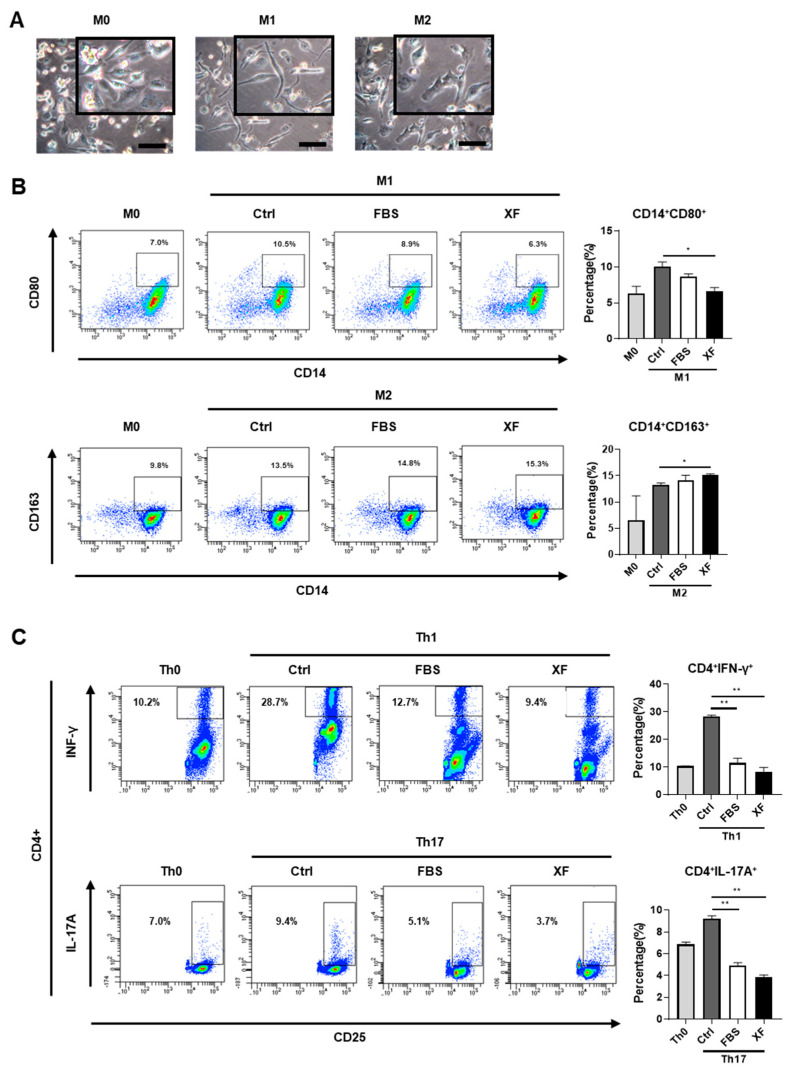
XF-MSCs regulate polarization of macrophages and T helper (Th) cells. (**A**–**B**) THP-1 cells were incubated with phorbol 12-myristate 13-acetate (PMA) for 24h and further overnight cultured in fresh medium for M0 differentiation. THP-1 derived macrophage (M0) was treated with M1 cytokines (IFN-γ, LPS) or M2 cytokines (IL-4, IL-13) and cultured with or without 10%-MSC (FBS) or XF-MSCs (XF) for 2 days. (**A**) Representative images of M0, M1, and M2. Scale bar, 100 µm. (**B**) After M1 and M2 induction, M1 (CD14^+^CD80^+^), M2 (CD14^+^CD163^+^) macrophage markers were measured by flow cytometry. (C) CD4+ T cells were cultured with specific lineage-driving cytokines with or without 10%-MSCs (FBS) or XF-MSCs (XF) for 5 days. The expression of Th1, Th17 specific markers (IFN-γ and IL-17A) from CD4+ T cells was determined by flow cytometry. The results are representative of independent experiments performed at least three times. (* *p* < 0.05, ** *p* < 0.01).

It has been reported that MSCs stimulate macrophages to produce anti-inflammatory cytokines such as IL-10, thereby inducing polarization into the M2 subtype [32,33]. To assess the immunomodulatory properties of XF-MSCs in macrophage polarization toward a pro-inflammatory or anti-inflammatory phenotype, PMA-stimulated macrophages were co-cultured with 10%-MSCs or XF-MSCs for 48 h in the presence of M1 cytokine or M2 cytokine. As shown in Figure 6A, THP-1-derived macrophages (M0) showed spindle/fibroblast morphology; M1 retained the spindle morphology, and there was a more spread morphology in M2. The induction of M1-type and M2-type macrophages was confirmed by phenotypic surface marker expression—CD80+ for M1-type and CD163+ for M2-type. Under XF conditions, the percentage of CD80+ M1 macrophages was significantly reduced compared to the control group (with M1 induction cytokines but no MSC co-culture), whereas the percentage of CD163+ M2 macrophages was increased (Figure 6B). These results indicate that XF-MSCs not only inhibited pro-inflammatory M1 activation but also induced anti-inflammatory M2 polarization comparable to the 10%-MSCs. To examine the effect of XF-MSCs on specific T cell subsets, helper T cells (Th) were generated by treating magnetically sorted CD4+ T cells from PBMNCs with lineage-driven cytokines in the presence of 10%-MSCs or XF-MSCs. Co-culture with either 10%-MSCs or XF-MSCs reduced the proportion of Th1 and Th17 cells compared to the control group (with Th induction cytokines but no MSC co-culture) (Figure 6C). These findings suggest that XF-MSCs could effectively suppress activated immunity by inducing anti-inflammatory M2 macrophage polarization and inhibiting Th1/Th17 differentiation. Therefore, XF-MSC can effectively substitute to 10%-MSC without the risk associated with the use of FBS.

### 3.6. XF-MSCs Improve Protective Effects against DSS-Induced Colitis in Mice

**Figure 7:**
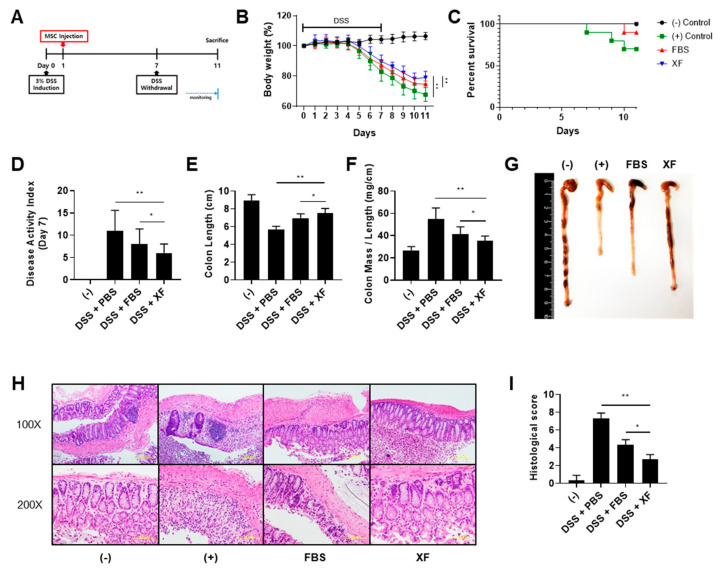
Administration of XF-MSCs increases the protective effects against dextran sulfate sodium (DSS)-induced colitis in mice. (**A**) Schematic presentation of experimental design. (**B**) The percentage of body weight loss and (**C**) the survival rate of mice were measured until day 11. On day 7, (**D**) disease activity index for colitis severity was measured as clinical progression. Mice were sacrificed 11 days after induction of colitis with 3% DSS, and (**E**,**G**) the colon length, (**F**) weight-to-length ratios of the colons were measured. *n* = 7~10 mice per group. (H) Colon sections were stained with H&E and (**I**) histopathologic evaluation was carried out by determining lymphocyte inflammation and crypt damage degrees. (−): negative control group, (+): DSS-administered group, FBS: WJ-MSCs cultured in FBS conditions, XF: WJ-MSCs cultured in XF conditions. (* *p* < 0.05, ** *p* < 0.01).

We explored whether the systemic administration of WJ-MSCs rescued mice from dextran sulfate sodium (DSS)-induced colitis and whether XF culture conditions enhanced the protective effect of WJ-MSCs against colitis (Figure 7A). The mice treated with 3% DSS solution exhibited clinical signs of colitis, including sustained weight loss, diarrhea, and bloody stools [34]. Intraperitoneal injections of WJ-MSCs ameliorated body weight loss and increased the survival rate of mice compared to the positive control group (Figure 7B,C). The administration of XF-MSCs restored the body weight of mice with DSS-induced colitis to around 80% that of the negative control mice and rescued 100% of the mice from colitis-induced lethality. In addition, the severity of disease activity index (DAI) scores for items such as stool consistency and bloody diarrhea was significantly suppressed in the XF-MSC-treated mice (Figure 7D). To determine the inflammatory state, the mice were euthanized on day 11 and length, mass weight, and histopathology of the colon were evaluated. The gross findings showed that the colon length was moderately restored by treatment with 10%-MSCs and further improved by treatment with XF-MSCs (Figure 7E,G). An increased colon mass/length ratio, which reflected colon wall thickening and rigidity, hyperemia, and evidence of colonic adhesions, was seen in the colitis-induced mice. This increased colon mass/length ratio was significantly reduced in the XF-MSC-treated mice compared to the 10% MSC-treated mice (Figure 7F). Histological examination of the colon of the DSS-treated mice showed severe submucosal thickening, destruction of the entire epithelium, and severe inflammatory cell infiltration in the lamina propria and submucosa (Figure 7H). In 10% MSC-treated mice, mucosal destruction and edema in the submucosa were reduced, and the administration of XF-MSCs greatly recovered the histological damage. The XF-MSC-treated mice also showed significantly reduced histological colitis scores, which indicated the extent of bowel wall thickening, crypt damage, and the infiltration of inflammatory cells (Figure 7I). Thus, these results indicate that XF-MSCs more effectively protected against intestinal inflammation in DSS-induced colitis than the 10%-MSCs.

## 4. Discussion

The therapeutic potential of MSCs is attributed to their immunomodulatory properties, mainly through cell–cell contact and the secretion of bioactive cytokines and chemokines, which restore homeostasis in inflammatory and degenerative diseases. Many attempts have been made to enhance the therapeutic effect of MSCs by priming with pro-inflammatory cytokines or genetic manipulation [35,36]. However, the effect of priming on the induction of immunogenicity or the long-term tumorigenic potential of MSCs needs to be studied [37]. To minimize these potential risks and enhance the immunomodulatory potential of MSCs, we tried to optimize culture media conditions using serum/xeno-free media. This serum/xeno-free MSC culture technique can also significantly reduce the scientific and ethical problems associated with the use of animal serum, such as the risk of possible contamination from animal-derived toxins and serum batch-to-batch irregularity (i.e., the concentrations of attachment factors and growth factors vary greatly amongst suppliers and batches) [20,38].

To avoid these safety issues but pursue fully defined culture conditions, we used vitronectin as a coating matrix instead of commercial products such as CELLStart coating and Coating Matrix Kit (Thermo Fisher Scientific), which contain undisclosed components. In our experimental setting, Wharton’s jelly-derived MSCs were cultured in serum/xeno-free conditions supplemented with vitronectin coating for at least three consecutive passages before the functional assay was performed. The long-term culture of XF-MSCs and 10%-MSCs showed similar proliferation rates. However, the expression of CD146 (also known as melanoma cell adhesion molecule, MCAM) was significantly decreased in the XF-MSCs. The analysis of human bone marrow samples revealed that the expression of CD146 decreased upon aging [39] and in vitro passage resulted in a decrease in the percentage and intensity of CD146-positive cells [40]. This indicates that the long-term culture of XF-MSCs possibly induced an early senescence state without losing the proliferation potential. Furthermore, age-related alterations in MSCs have been reported to relate to a shift in differentiation from an osteogenic to an adipogenic potential [41,42,43]. Consistent with the decreased CD146 expression in XF-MSCs, the mutually exclusive lineage fate determinations of XF-MSCs toward enhanced adiopogenesis indicate possible aging-related molecular changes in the serum/xeno-free culture conditions. In the aspect of clonogenicity, no colonies were obtained in the CFU-F assay using xeno-free media, similar to a previous report [44]. As the cumulative population doubling level (CPDL) data showed comparable survival and proliferation rates in 10%-MSCs and XF-MSCs, cell-to-cell contact and related signals might be critical factors for XF-MSCs to form colonies.

Selich and colleagues used an interesting multicolor lentiviral barcode labeling approach to follow the clonal dynamics during in vitro MSC expansion from whole umbilical cord pieces [45]. In this experimental setting, the initial clonal composition with highly complex cell populations decreased markedly, and transiently dominating populations followed by a selection of single clones over time were observed. In this context, the environment of xeno-free culture conditions in the vitronectin-coated dish favored the selection of primitive MSCs, with enhanced adipogenic differentiation potential and immunomodulatory ability. Alternatively, xeno-free culture conditions possibly altered the physiologies of MSCs. The choice of attachment molecule and xeno-free medium may affect the selection of the desired MSC properties with high therapeutic efficacy. Further optimization of alternative attachment molecules or xeno-free media is needed.

We found that long-term serum/xeno-free culture conditions altered the molecular signature and functional properties of MSCs. First, primitive HSCs (CD34+CD90+ cells) were enriched in the XF-MSC co-culture, which expressed high levels of HSC niche-related genes, such as CXCL12 [46] and VCAM1 [47]. MSCs cultured in the serum/xeno-free culture system had advantages of mimicking the HSC niche, namely the maintenance and expansion of HSCs with superior phenotypic attributes. Second, the gene expression screening data suggested that IDO was significantly elevated in XF-MSCs stimulated by IFN-γ and TNF-α among a variety of genes that could potentially be associated with immunosuppressive functions. Indeed, human peripheral blood-derived T cell activation and proliferation were effectively suppressed by XF-MSCs compared to 10%-MSCs through IDO signaling, confirmed by the inhibitor study (Figure 5).

Several previous studies examined the effect of serum/xeno-free culture conditions on immunomodulatory function in MSCs. Yoshida et al. showed that serum-free, but not xeno-free, medium for MSCs (derived from bone marrow and adipose tissue) enhanced immunosuppressive and antifibrotic abilities by inducing a change in the phenotype of macrophages to M2 type through TSG-6 expression [29]. Consistent with these data, XF-MSCs induced the polarization of macrophages from a pro-inflammatory M1 to an immunosuppressive M2 phenotype. The expression level of TSG-6 or COX2 [33] was not elevated in the XF-MSCs. However, upregulated TGF-β expression possibly explains the M1/M2 shift, as a recent study reported that TGF-β secreted by MSCs could skew macrophage polarization toward an M2-like phenotype via the Akt/FoxO1 pathway [48]. In contrast, Patrikoski et al. demonstrated that all groups of MSCs derived from adipose tissue (AD-MSCs) had weak immunogenicity regardless of FBS- or XF culture conditions [28]. However, stronger suppression was observed in cells expanded in FBS conditions, whereas higher cell numbers were required in order to display suppression in the XF culture conditions. As we observed a lack of CFU-F ability in low-density cultures of XF-MSCs, further study is needed to reveal the physiological role of cell density and cell adhesion molecules in XF culture conditions. However, these studies did not demonstrate the comprehensive immunomodulatory properties of MSCs in the aspects of T cell proliferation and Th/macrophage polarization.

To study the physiological properties of the altered molecular signature in XF-MSCs, we tested the effects of XF-MSCs on other immune cells in both in vitro and in vivo settings. As IDO secreted from MSCs led to tryptophan depletion and the subsequent inhibition of allogeneic T cell responses [49], XF-MSCs with higher IDO levels showed the modulation of IFN-γ-producing Th1 cells as well as IL-17-secreting Th17 cells, in a shift from pro-inflammatory to anti-inflammatory conditions. Furthermore, XF-MSCs showed an enhanced ability to suppress M1-type macrophages and induce M2-type macrophages, possibly through elevated IDO expression [32]. These in vitro findings were further linked to the therapeutic effect of XF-MSCs on an experimental colitis model with improved survival rate and reduced disease activity. The enhanced protective effect against the XF-MSC colitis model was possibly due to the facilitation of monocyte differentiation into anti-inflammatory M2-type macrophages as well as the direct inhibition of Th1 cells in the colitis model. Taken together, these results indicate that serum/xeno-free culture conditions induced enhanced IDO production by human WJ-MSCs, which led to efficient anti-inflammatory modulation of immune cells, and the immunosuppressive effects of XF-MSCs are sufficient for the significant attenuation of colitis. Our results suggest that the use of serum/xeno-free cultured MSCs can be a safe and effective therapeutic alternative to the cell-based treatment of inflammatory bowel disease.

## Figures and Tables

**Table 1:** Primer sequences.

Name	Primer Direction	Sequences
PPAR-γ	Frw	CCTCCGGGCCCTGGCAAAAC
	Rev	CTCCTGCACAGCCTCCSCGG
aP2	Frw	GGGTCACAGCACCCTCCTGA
	Rev	GGTTTGGCCATGCCAGCCAC
runx2	Frw	CCCAGTATGAGAGTAGGTGTCC
	Rev	GGGTAAGACTGGTCATAGGACC
ALP	Frw	ATGTCATCATGTTCCTGGGAGAT
	Rev	TGGTGGAGCTGACCCTTGAG
COMP	Frw	AGCAGATGGAGCAAACGTATTG
	Rev	ACAGCCTTGAGTTGGATGCC
Collagen XIa1	Frw	CGGAGGCAAACATCGTTGAT
	Rev	ATTTGGCTCATTTGTCCCAGAA
COX2	Frw	AGACGCCCTCAGACAGCAAA
	Rev	TCCTGTCCGGGTACAATCGC
IDO	Frw	CCTGAGGAGCTACCATCTGC
	Rev	TCAGTGCCTCCAGTTCCTTT
TGF-β1	Frw	GATGTCACCGGAGTTGTGCG
	Rev	GCCGGTAGTGAACCCGTTGAT
IL-1β	Frw	CTCTTCGAGGCACAAGGCAC
	Rev	CAAGTCATCCTCATTGCCACTGT
IL-18	Frw	ACTGCCTGGACAGTCAGCAA
	Rev	GCAGCCATCTTTATTCCTGAGA

PPAR-γ, Peroxisome proliferator-activated receptor gamma; aP2, adipocyte protein 2; runx2, Runt-related transcription factor 2; ALP, Alkaline phosphatase; COMP, Cartilage oligomeric matrix protein; COX2, Cyclooxygenase-2; IDO, Indoleamine-pyrrole 2,3-dioxygenase; TGF-β1, Transforming growth factor beta 1; IL-1β, Interleukin 1 beta; IL-18, Interleukin-18.

## Supplementary Materials

The following are available online at https://www.mdpi.com/2077-0383/9/9/2913/s1, Figure S1: Cell surface markers of WJ-MSCs cultured in FBS and XF conditions; Figure S2: Colony-forming unit-fibroblast (CFU-F) assay of WJ-MSCs cultured in FBS and XF conditions; Figure S3: Track cell division in the distinct compartments using CFSE staining; Figure S4: Regulation of the immunosuppressive effect of XF-MSCs by IDO.
