# Supplementary material for: Xeno-Free Condition Enhances Therapeutic Functions of Human Wharton’s Jelly-Derived Mesenchymal Stem Cells against Experimental Colitis by Upregulated Indoleamine 2,3-Dioxygenase Activity
Source: J Clin Med. 2020 Sep 10;9(9):2913. doi: 10.3390/jcm9092913 (PMC7565923; doi:10.3390/jcm9092913)
Supplement: Supplementary file 1 [file jcm-09-02913-s001.pdf]

Supplementary Figure 1

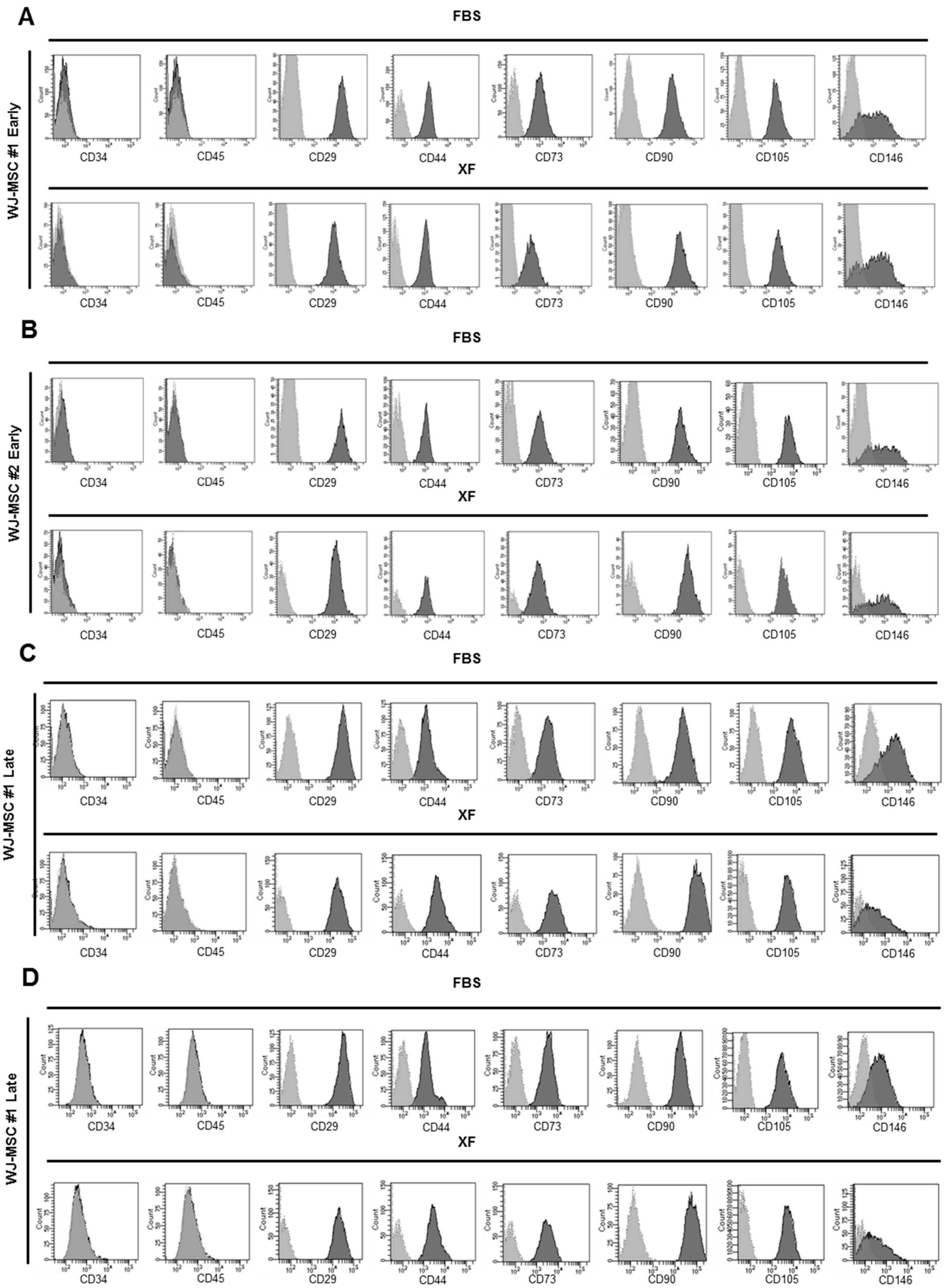

Supplementary Figure 1. Cell surface markers of WJ-MSCs cultured in FBS and XF conditions.

Cell surface markers of WJ-MSCs cultured in FBS and XF conditions were measured by flow cytometry for the expression of the MSCs specific antigen CD29, CD44, CD73, CD90, CD105, CD146 and CD34, CD45.

Supplementary Figure 2

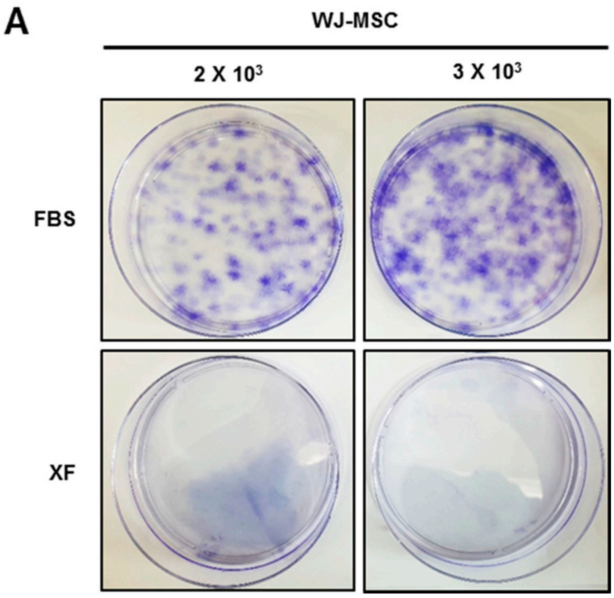

Supplementary Figure 2. Colony-forming unit-fibroblast (CFU-F) assay of WJ-MSCs cultured in FBS and XF conditions.

(A) WJ-MSCs cultured in FBS or XF conditions were seeded in 100mm culture dish and then stained with 1% crystal violet 14 days.

Supplementary Figure 3

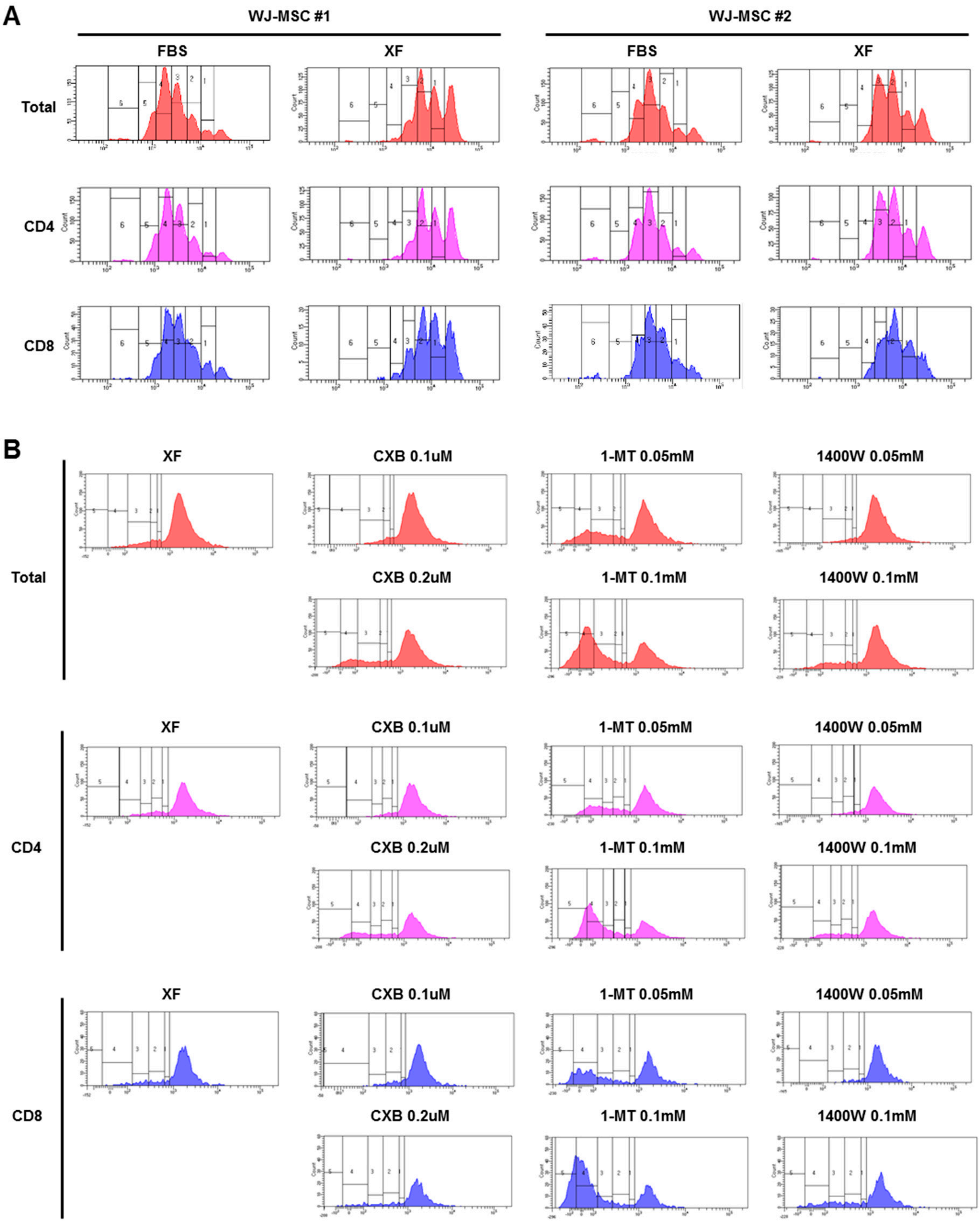

Supplementary Figure 3. Track cell division in the distinct compartments using CFSE staining.

(A) Activated T cells were cultured with 10%-MSCs or XF-MSCs for 6 days. T subsets were stained with anti-CD3, anti-CD4, and anti-CD8 and analyzed by flow cytometry. The fluorescence profile of CFSE-labeled cells was identified by six divisions. (B) After treating XF-MSCs with the celecoxib, 1-MT or 1400W at the indicated dose, the cells were incubated with activated T cells. Proliferation of the total T, CD4+ T and CD8+ T cells was measured by flow cytometry, and the fluorescence profile of CFSE-labeled cells was identified by five divisions. The results show representative of independent experiments performed at least three times.

Supplementary Figure 4

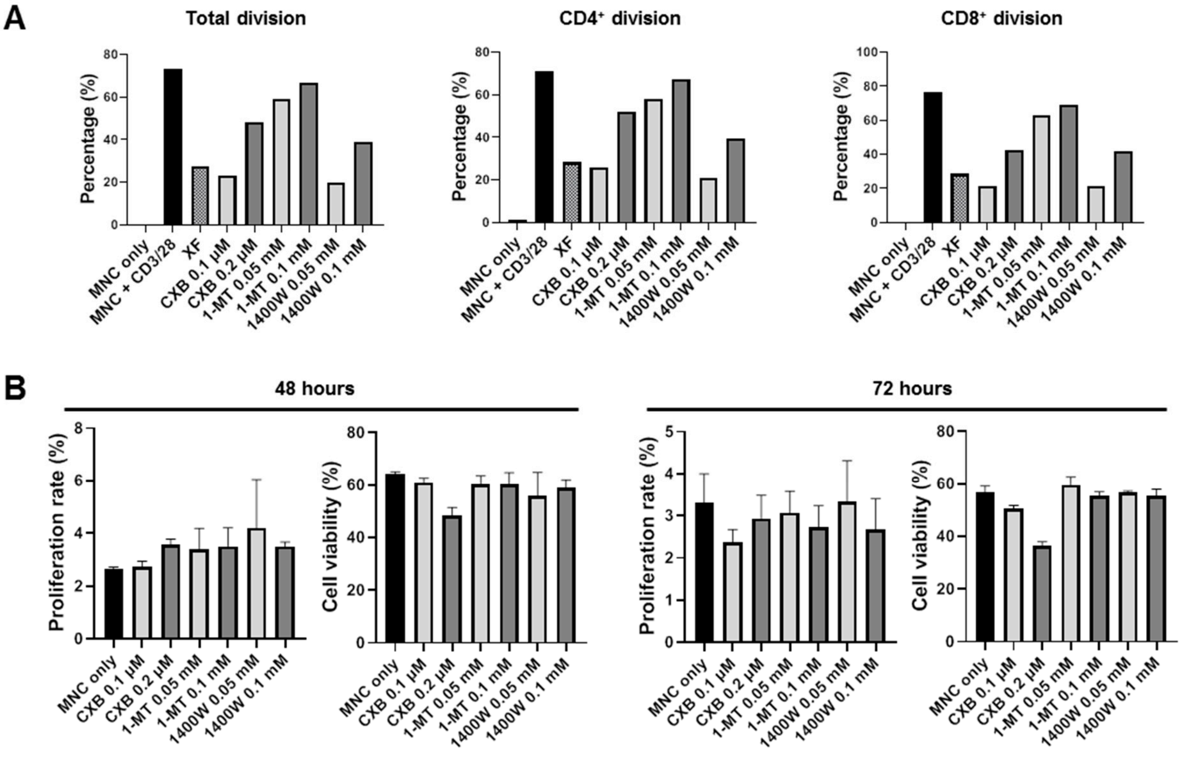

Supplementary Figure 4. Regulation of the immunosuppressive effect of XF-MSCs by IDO.

(A) After treating XF-MSCs with the celecoxib, 1-MT or 1400W at the indicated dose, the cells were incubated with activated T cells (PBMCs donor#2). Proliferation rate of the total T, CD4 + T and CD8+ T cells was quantified. (B) CFSE-labeled PBMCs were treated with the celecoxib, 1-MT or 1400W for 48 and 72 hours, and then stained with 7AAD. Flow cytometry was determined to assess PBMCs proliferation and cell viability
